# “Researcher” bias: How our assumptions on technology affect research of older adults

**DOI:** 10.3389/fpubh.2022.1034497

**Published:** 2022-11-02

**Authors:** Catherine A. Clair, Tonisha J. Melvin, Janiece L. Taylor, Martha Abshire Saylor

**Affiliations:** ^1^Department of Health, Behavior and Society, Johns Hopkins Bloomberg School of Public Health, Baltimore, MD, United States; ^2^Johns Hopkins School of Nursing, Baltimore, MD, United States

**Keywords:** older adults, technology, assumptions, person-centered, bias

## Introduction

As researchers, we are comfortable with different aspects of the scientific method: Designing studies, implementing interventions, collecting and analyzing data, and synthesizing results. We progress in our careers, solidifying our skills and potentially, our assumptions.

Assumptions are human. Assumptions are heuristics that allow us to more efficiently operate within our natural world (1). However, if we do not actively engage with and question our assumptions, we become comfortable with our own biases.

For those with careers in the field of gerontology, we have undoubtedly heard comments like, “You won't get good data by doing interviews with older adults over the phone.” or “Older adults will not participate in research if it involves technology.” These assumptions—that older adults cannot operate technology or cannot answer questions over the phone or on Zoom—reflect not only ageism but a scientific bias (2).

Yet, comfort with technology exists on a spectrum (3, 4). We know that many older adults are comfortable with technology, such as smartphones and tablets (5). Adoption of technology has accelerated, particularly due to the COVID-19 pandemic. One study found using data from the National Health and Aging Trends Study reported that older adults were engaging in more technology-based activity and technology-based health communication (6). At the same time, we also know that nearly 22 million older adults in the United States lack broadband access in their home (7).

While our assumptions about older adults and technology may be based in reality, we risk limiting ourselves and our research if we let our assumptions take the reins.

We believe that assumptions regarding older adults and their ability to access and use technology tend to be based on pre-COVID experiences and limit our study designs and approaches. We recommend that researchers consider the needs and abilities of their specific older adult population regarding technology and respond proactively to the specific experiences of their participants.

## What we can learn from our participants

It is important as researchers that we do not make assumptions for our participants but instead follow their lead. We can learn from community-based participatory research principles, particularly the collaborative involvement of participants in all phases of the research, to bring the voice of older adults into our research (8). While this sense of openness and flexibility may cause some anxiety, we can ask our participants at various stages of our research about how to meet their needs and improve the quality of our science (9). We share two case studies below representing reflections of our own biases regarding older adult populations and technology and the how our team(s) reframed their thinking.

### Case study 1: Older African American women living with pain and low mood

We are conducting a study testing a behavioral, pain-management intervention for older African American women living with pain and low mood. During intervention development, we explored preferences for visit setting. The team assumed that these women would prefer virtual visits, particularly during the COVID-19 pandemic due to safety and health concerns, and would have the capability to access the intervention virtually. Both in-person and virtual options were presented to the participants; so far, of twenty participants, twelve have opted to do in-person visits. When asked why, one participant said that having the nurse come to her house was “rewarding, (especially) when you live alone.” Another participant commented that she did not have Internet access in her home and would have been unable to participate in a virtual-only intervention.

### Case study 2: Caregivers of persons living with heart failure

In another study we are testing a self-care intervention for caregivers of persons living with heart failure. During a pilot phase of the study, the protocol was written for in-person visits as it was assumed this would provide the easiest and most person-centered means of delivering the intervention: The participant would not have to travel, the nurse interventionist would gain more context to aid with intervention delivery. The team also assumed that older caregivers would not “get as much” from virtual intervention visits. Due to the pandemic, the pilot study went entirely virtual. In an effort to support equitable participation, tablets were provided to all participants. However, all participants had their own technological device, and the tablets, though provided, were not utilized. When asked about the change in modality, participants had no issue using their own technology and preferred the flexibility that virtual meetings had to offer: Virtual visits could be canceled and rescheduled more easily. In fact, the virtual visits were person- and caregiver-centered.

These two stories have commonalities between them. The decisions made during the study design stage were intended to reduce study burden on the participants; however, some assumptions were made regarding the population's ability to access and use technology. Yet the most important takeaway from these case studies is the responsiveness of the researchers. Listening and responding to participant feedback is key to delivering participant-centered research.

## We are human

Now, as mentioned above, making assumptions is a natural human response. Often, our assumptions are informed by years of practice and research, previously published literature, and communication with colleagues and peers in our fields. Making assumptions is reasonable. However, by making these assumptions related to technology, do we limit the “presumed” benefit of our research or interventions? Do we limit our potential findings? And do we limit ourselves as researchers in terms of creativity and expansive thought?

## Discussion

We propose choices that researchers can make to curb our natural instincts regarding our research and study design (Table 1). As research continues to evolve, let us ask ourselves:

1. Is there flexibility in our protocol? For example, offering in-person and virtual options for intervention delivery and/or data collection. For older adults, in-person visits may be challenging given mobility limitations and transportation options (10, 11); in contrast, virtual visits may be challenging given access and comfort with using technology (3, 7).
2. Are the decisions we are making participant-driven? For example, if our older adult participants have stated preference to a certain modality of intervention delivery and/or data collection, we should be responsive when drafting or revising the protocol (12).
3. Is there opportunity for us to receive feedback? For example, including post-intervention interviews or surveys with older adult participants to ask about intervention delivery and/or data collection modalities (13–15). We want to make their participation “well-worth the effort” (14).

**Table 1:** Our guiding questions and proposed solutions.

**Guiding questions**	**Solutions to our assumptions**
Flexibility in protocol & pre-study activities	• Meet with your IRB representative to discuss opportunities for flexibility in your protocol. • Include a community member/member of your population of interest as a member of the study team. • Actively seek to create a diverse study team in terms of gender, race, ethnicity, age, career, etc.
Participant-driven design	• Incorporate elements of human-centered design into your protocol and interventions. • Create a Patient & Family Advisory Council to guide your study team. • Create a community advisory board to guide your study team.
Opportunity for feedback	• Utilize qualitative, quantitative, and/or mixed methods evaluation for richer understanding of your participants' experiences. • Include a participant experience survey or interview after completion of the research. • Use an open-label pilot design prior to the full intervention. • Talk to experts outside of your comfort zone for their thoughts and advice.

It is important to note that our assumptions are not always wrong, and we make them for different reasons: Efficiency, history, etc. But as researchers, we have to constantly question our assumptions, especially those regarding technology and different older adult populations. This active questioning allows us to minimize the “researcher” bias that we may introduce into our studies. We cannot eliminate all bias in our science, but we can continually challenge our assumptions by asking questions and elevating the voice of the older adult communities we serve.

## Author contributions

All authors listed have made a substantial, direct, and intellectual contribution to the work and approved it for publication.

## Conflict of interest

The authors declare that the research was conducted in the absence of any commercial or financial relationships that could be construed as a potential conflict of interest.

## Publisher's note

All claims expressed in this article are solely those of the authors and do not necessarily represent those of their affiliated organizations, or those of the publisher, the editors and the reviewers. Any product that may be evaluated in this article, or claim that may be made by its manufacturer, is not guaranteed or endorsed by the publisher.
